# The Protective Effects of Fasciotomy on Reperfusion Injury of Skeletal Muscle of Rabbits

**DOI:** 10.1155/2017/7238960

**Published:** 2017-08-10

**Authors:** Rui-Hua Li, Jin Li, Shi-Lian Kan, Xi-Nan Zhang

**Affiliations:** ^1^Department of Hand and Microsurgery, Tianjin Hospital, Tianjin 300211, China; ^2^Orthopaedics Integration of Traditional Chinese Medicine with Western Medicine, Tianjin University of Traditional Chinese Medicine, Tianjin 300193, China

## Abstract

The authors aim to investigate protective effects of fasciotomy against ischemia reperfusion injury of skeletal muscle in rabbit and to compare the treatment effects of prereperfusion + fasciotomy and fasciotomy + postreperfusion against ischemia reperfusion injury of skeletal muscle. 24 healthy male Japanese white rabbits were randomly divided into 3 groups, and 4 hours' ischemia was established in these rabbits through surgery. Six hours' reperfusion was performed in group A; reperfusion + postfasciotomy was performed in group B; and prefasciotomy + reperfusion was performed in group C. Result showed that prefasciotomy and postfasciotomy could protect skeletal muscle against ischemia reperfusion injury, reduced MDA (malondialdehyde) expression, MPO (myeloperoxidase) expression, and apoptosis of muscle in the reperfused areas, increased Bcl-2 expression, and decreased Bax expression. The MDA and MPO levels in group B and group C were significantly lower than those in group A, and MDA and MPO levels in group C were significantly lower than those in group B. Prefasciotomy and postfasciotomy could protect against ischemia reperfusion injury in skeletal muscle. The protective effects of prefasciotomy against ischemia reperfusion injury are better than postfasciotomy.

## 1. Introduction

With the accelerated development of productive forces and transportation, the occurrence of severed extremity increased obviously. The severed upper extremity is a dangerous trauma for orthopedics because the severed limb has much muscle tissue and limited time to tolerate ischemia. The replantation of severed upper extremity has great risk, and the functional recovery is not satisfactory [1, 2]. With prolonging the time of limb ischemia, the released oxygen free radicals increased after reperfusion [3]. Recovery of blood flow after reperfusion cannot improve the function of the muscle cells but will cause serious injury [4]. In clinical work, trauma, arterial embolism, primary thrombosis, artery transplantation, replantation, compartment syndrome, and longtime application of tourniquet all could cause serious skeletal muscle ischemia and subsequent reperfusion injury, which affects the survival of patients with severed extremity and even cause limb amputation [5–7].

Some progress has been made in recent studies about the reperfusion injury after limb ischemia. Scholars proposed some methods and suggestions in the prevention and treatment of reperfusion injury after ischemia of skeletal muscle, but they are rarely used in the current clinical treatment [8–10]. Current proposed prevention mechanism/methods about reperfusion injury of skeletal muscle ischemia are exogenous protection method and the induced endogenous mechanism in body. Exogenous protective methods are mainly using drugs to prevent and treat reperfusion injury. Murry et al. [11] proposed the endogenous protection mechanisms of ischemia reperfusion injury through the study of ischemic preconditioning: the tissues could tolerate or attenuate relative longtime ischemia reperfusion injury after several times of transient ischemia. The protective effect of this endogenous mechanism in body has been confirmed in human and different species of animals. A study demonstrated that three times' repeated 10 minutes of ischemia followed by reperfusion for 10 minutes can significantly reduce the subsequent ischemia reperfusion injury induced by longtime ischemia. The protective effect of ischemic preconditioning is correlated with the frequency of ischemic preconditioning, but the reasonable time interval and effective management cycle number of the pretreatment have not yet been determined [12, 13]. The pathophysiology of ischemic preconditioning for skeletal muscle might be due to improving impaired electron transport chain and oxidative phosphorylation in ischemic skeletal muscle [14]. The supposed molecular mechanism of tissue protection of ischemic postconditioning involved the inhibition of opening of mitochondrial permeability transition pores (mPTP) [15]. Previous studies also demonstrated that postconditioning could decrease systemic inflammatory response (TNF-*α*) and cause a marked reduction in reperfusion-related organ dysfunctions (lungs and kidneys) in a model of partial ischemia using infrarenal aortic clamping [16].

Reperfusion injury after serious limb ischemia can cause severe edema in tissues, increase bone compartment pressure, increase the necrosis of muscle, and influence functional recovery. And fasciotomy can effectively alleviate ischemia reperfusion injury of limb. Some researcher proposed that fasciotomy can be performed in severe limb ischemia replantation and vascular anastomosis surgery. Routine forearm fasciotomy was performed in the upper arm replantation of our surgery. Oxygen free radicals with high activity and cytotoxicity damage cell through lipid peroxidation in ischemia reperfusion injury of skeletal muscle [17, 18]. The determination of MDA (malondialdehyde) can indirectly reflect the level of oxygen radicals. The neutrophils of white blood cells play important roles in ischemia reperfusion injury of skeletal muscle. MPO (myeloperoxidase) is a blood protein which exists in neutrophil azurophilic granules and macrophage cells; level of MPO could reflect the infiltration degree of neutrophils in damage zone of tissue. In this study, we established severed extremity in rabbit and compared the apoptosis degree of skeletal muscle after ischemia reperfusion treated by prefasciotomy and postfasciotomy, to provide useful reference for the prevention and treatment of reperfusion injury of skeletal muscle.

## 2. Methods

### 2.1. Animal Preparation

**Figure 1:**
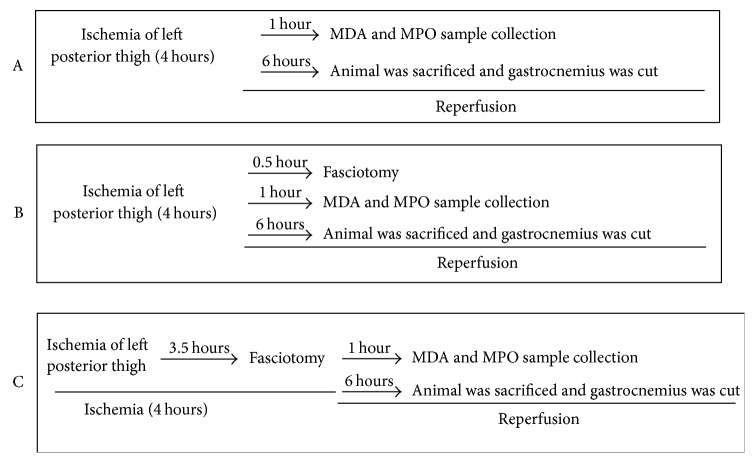
The flowchart of experiment.

Male adult Japanese white rabbits (SPF level), weighing 2000 grams–2250 grams, were purchased from experimental animal center of Military Medical Science Academy of the People's Liberation Army. After being fed for 2 weeks, 24 rabbits were randomly divided into 3 groups with 8 in each group: group A: ischemia reperfusion group, 4 hours' ischemia following 6 hours' reperfusion in rabbits; group B: reperfusion + postfasciotomy group, where fasciotomy was performed after 0.5 hours' perfusion in ischemic rabbits, then following another 5.5 hours' reperfusion; and group C: prefasciotomy + reperfusion group, where fasciotomy was performed 0.5 hours before the end of 4 hours' ischemia followed by 6 hours' reperfusion. The detailed grouping and sample collected time points are listed in Figure 1. The experiment protocol was reviewed and approved by the Animal Management Committee of Tianjin Hospital.

### 2.2. Ischemia Reperfusion Injury Model of Skeletal Muscle

**Figure 2:**
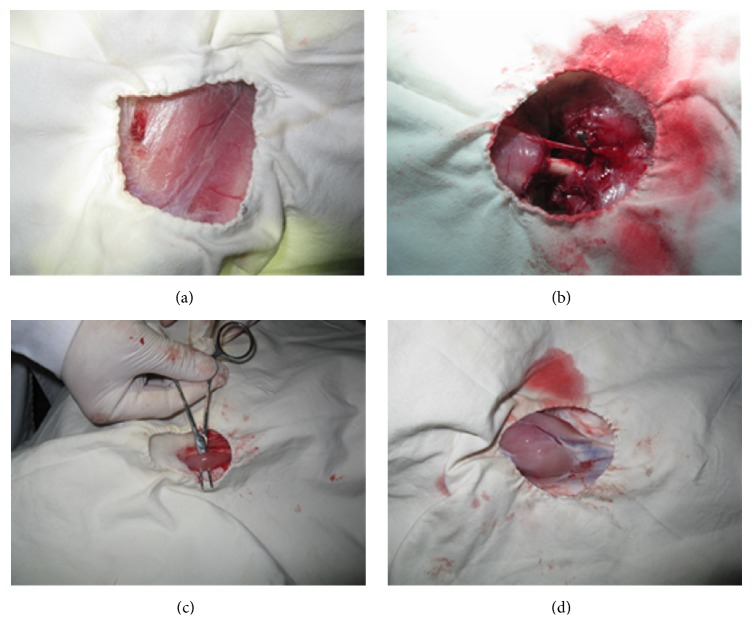
Ischemia reperfusion injury model and reperfusion preparations in rabbit. (a) Exposure of femoral artery and vein; (b) clamping of femoral artery and vein; (c) exposure of musculi hippicus; (d) exposure of gastrocnemius muscles.

Rabbits were anaesthetized by 0.5% pentobarbital intraperitoneally with dose of 50 mg/kg body weight. After the successful anesthesia, rabbits were fixed on the board in a supine position. The central skin slightly above left posterior thigh of rabbit was cut horizontally after disinfection (Figure 2(a)). All the rear thigh muscles to rabbit femoral shaft were cut off. The sciatic nerve was also cut off. The muscle and skin of the rear femoral shaft were completely cut off and then sutured in situ.

The rabbit was put on dorsal position. The upper skin of the thigh middle was cut horizontally until the rear thigh. The 1/3 femoral vein of left thigh was separated and protected under the operating microscope. All the muscle and femoral nerves of left thigh were transected. The muscle of anterior thigh was sutured in situ. Only femoral vein and bone scaffold of the left thigh were connected with rabbit body connection. The femoral arteriovenous vascular was clamped (Figure 2(b)) and clamped location was changed every hour to avoid longtime compression of vascular wall caused by vascular injury. Intraperitoneal anesthesia was performed 4 hours after ischemia. The incision of left anterior thigh was cut and vascular clamp was removed. The postprocessing operations were performed and the blood recovery was observed under surgical microscope. The incision was sutured after the recovery of artery blood flow.

### 2.3. Measurement of Malondialdehyde (MDA) and Myeloperoxidase (MPO)

1 ml venous blood in rabbit's right thigh (without operation thigh) was extracted after 1 hour's reperfusion. The blood was put still for 1 hour and then centrifuged at 2500 rpm for 15 min. The serum was preserved in the −70°C. The skeletal muscle samples in the tibialis anterior muscle of front limb leg (Figure 2(c)) were harvested at the same time point after animal was anaesthetized again. Levels of MDA and MPO were determined by double antibody sandwich method. Briefly, purified rabbit MDA/MPO antibodies were coated onto a microplate, the standard and test samples; MDA and MPO were added to micropores coated with rabbit monoclonal antibody, connected with horseradish peroxidase (HRP) labeled MDA/MPO antibody, washed, and colored by 3,3′,5,5′-tetramethylbenzidine (TMB). The absorbance value (OD value) in the 450 nm wavelength was measured. The standard curve was drawn to calculate the sample concentration of MDA/MPO in rabbits (MDA/MPO Kit, Jiancheng Biotech, Nanjing, CHN); results are expressed as nmol/ml (MDA) and U/L (MPO).

### 2.4. Measurement of Muscle Death Degree

The succinic dehydrogenase is inactive and cannot separate hydrogen binding with tetrazolium salts and coloring. The survival and death of muscle tissue can be distinguished according to the color. The rabbits were sacrificed by decapitation after 6 hours' reperfusion, gastrocnemius at the severed limb was cut completely (Figure 2(d)). The fresh resected specimens of gastrocnemius were frozen at −20°C for 30 minutes and cut into 6 equal pieces along with the long axis of gastrocnemius muscle. The muscle piece was cultured in 0.05% NBT (nitro blue tetrazolium) solution (nitroblue tetrazolium + 0.2% TRIS buffer at PH7.4) at 37°C for 30 minutes. The survived muscle shows purple blue color, while the inactivated muscle tissue shows red color. The stained muscle tissue was photographed with a digital camera directly and analyzed by Image-pro express 10 software (Cybernetics Media, Inc.). The percentage of death muscle equals red area of muscle/total area of muscle in 6 muscle pieces.

### 2.5. Detection of Cell Apoptosis

1/4 of proximal gastrocnemius tissue was collected, fixed by formaldehyde, made transparent by dimethylbenzene, and embedded in paraffin routinely to prepare serial sections of 5 *μ*m. Routine hematoxylin and eosin (HE) staining was performed to observe the apoptosis of cells. The apoptosis of skeletal muscle cells was measured by TUNEI method. BI 2000 medical image analysis software (Techman Software Ltd., Chengdu, CHN) was used to analyze gastrocnemius muscle tissue. Six visions were selected randomly in each slice at low magnification; the average absorbance of each slice was calculated and obtained. Positive reacting nuclei in 100 randomly selected cells were calculated.

### 2.6. Bax and Bcl-2 Expression

Both Bax and Bcl-2 are important apoptosis mediators but play opposite roles. Bax is proapoptotic and BCL-2 is considered as antiapoptosis mediators. In the current study, expressions of Bax and Bcl-2 were determined by immunohistochemistry method. Briefly, sections were dewaxed. Muscle sections were then incubated with the primary antibodies overnight at 4°C. Primary antibodies included rabbit polyclonal antibody to Bcl-2 and rabbit monoclonal antibody to Bax from Santa Cruz Biotechnology (Santa Cruz, CA, USA). The antibodies were used at dilutions of 1 : 50 for Bcl-2 and Bax. Then, the sections were incubated with two-step immunohistochemistry detection reagent (PV6001 and PV6002; Zhongshan Golden Bridge, Beijing, China) at 37°C for 30 minutes. A positive brown color appeared in the slices after 3,39-diaminobenzidine colorization. According to the distribution of positive cells in each section, we selected 5 different visions and calculated the average absorbance value of Bax and Bc1-2 using Image-Pro Plus image analysis system (Media Cybernetics, Rockville, MD, USA).

### 2.7. Statistical Analysis

All obtained data were analyzed by SPSS11.0 statistical analysis software package (IBM, Chicago, IL, USA). The experimental data were expressed as mean ± standard deviation. Wilcoxon rank sum test was used for 2-group comparison; ANOVA was used for multigroup comparison. *p* < 0.05 was considered as statistically significant.

## 3. Results

### 3.1. Death Degree of Skeletal Muscle

**Figure 3:**
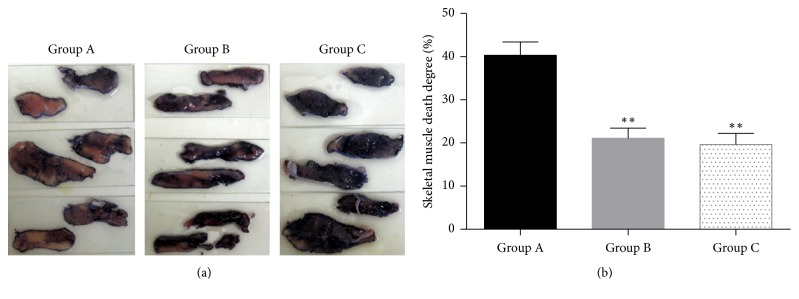
Death degree of skeletal muscle: the survival and death of muscle tissue can be distinguished according to the color. (a) Resected muscles in each group. (b) Comparison results of death degree after image analysis.

The survival and death of muscle tissue can be distinguished according to the color (Figure 3(a)). Compared with rabbits which received ischemia reperfusion (group A), the death degree of skeletal muscle in reperfusion + postfasciotomy group (group B) and prefasciotomy + reperfusion group (group C) was alleviated greatly (*p* = 0.0082 and 0.0032, resp., compared with group A). The death of skeletal muscle was slightly alleviated in group C compared with group B but has no statistical difference (*p* = 0.1002, Figure 3(b)).

### 3.2. Apoptosis Degree of Skeletal Muscle Cell

**Figure 4:**
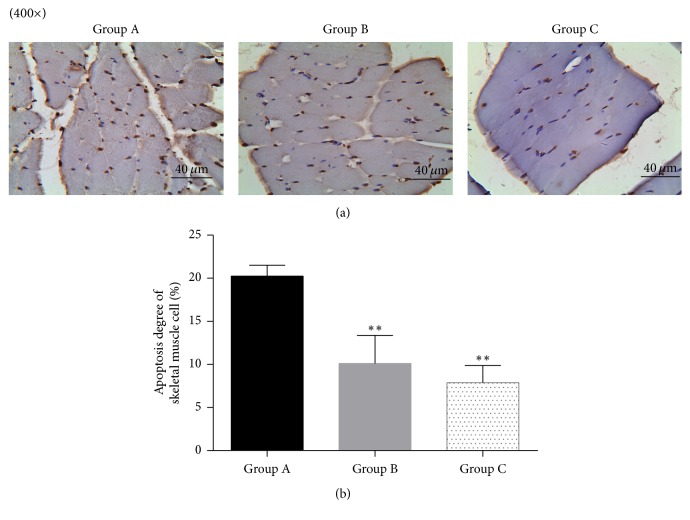
Apoptosis degree of skeletal muscle cell. (a) HE staining in each group. (b) Absorbance value comparison in each group. ^*∗∗*^*p* < 0.01, compared with group A.

TUNEI staining showed that apoptotic nuclei were brown, with irregular shape. The apoptotic nuclei size is not consistent. While the nuclei of normal cells were counterstained with hematoxylin blue, the nucleus is relatively large with regular shape and uniform size. In addition, HE staining (Figure 4(a)) showed that the gap between apoptotic cells increased significantly in ischemia reperfusion (group A), while the gaps were significantly deceased in reperfusion + postfasciotomy group (group B) and prefasciotomy + reperfusion group (group C) (*p* = 0.0098 and 0.0021 resp., compared with group A). The gap was relatively tight in group C compared with group B, which demonstrated that the apoptosis rate of skeletal muscle cell was lower in group C compared with group B. However, comparison of absorbance value showed that there was no statistical different between the 2 groups (*p* = 0.0604, Figure 4(b)).

### 3.3. Expression of MDA and MPO

**Figure 5:**
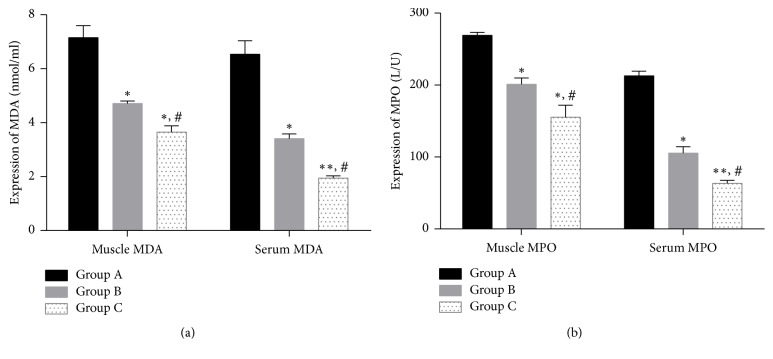
Expression of MDA and MPO. (a) MDA and (b) MPO. ^*∗*^*p* < 0.05, ^*∗∗*^*p* < 0.01, compared with group A. ^#^*p* < 0.05, compared with group B.

The expressions of MDA and MPO in serum and skeletal muscle were significantly higher in reperfusion + postfasciotomy group (group B) and prefasciotomy + reperfusion group (group C) compared with ischemia reperfusion group (group A) (all *p* < 0.05, Figure 5(a)). In addition, the expressed MDA in serum and skeletal muscle were significantly higher in prefasciotomy + reperfusion group (group C) than reperfusion + postfasciotomy group (group B) (*p* = 0.049 in muscle and *p* = 0.0154 in serum, resp.). The expressions of MPO in serum and skeletal muscle (Figure 5(b)) were also decreased furtherly in group C when compared with group B (*p* = 0.044 in muscle and *p* = 0.0396 in serum, resp.).

### 3.4. Expression of Bax and Bcl-2 in Skeletal Muscle

**Figure 6:**
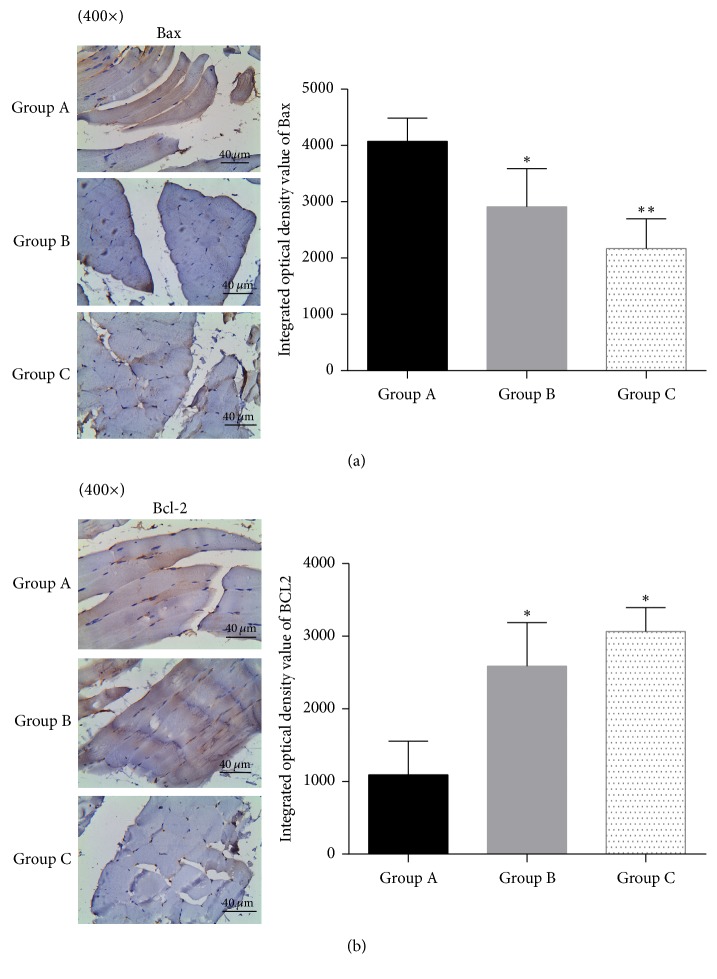
Expression of Bax and Bcl-2 in skeletal muscle by immunohistochemistry analysis. (a) Expression of Bax. (b) Expression of Bcl-2. ^*∗*^*p* < 0.05, ^*∗∗*^*p* < 0.01, compared with group A.

Comparison of integrated optical density in immunohistochemistry section showed that expression of Bax was significantly lower in reperfusion + postfasciotomy group (group B) and prefasciotomy + reperfusion group (group C) compared with ischemia reperfusion group (group A) (*p* = 0.0144 and 0.0002, resp., compared with group A), and the expression was lower in group C compared with group B but has no statistical difference (*p* = 0.0895, Figure 6(a)). The expression of Bcl-2 was significantly higher in reperfusion + postfasciotomy group (group B) and prefasciotomy + reperfusion group (group C) compared with ischemia reperfusion group (group A) (*p* = 0.0432 and 0.0324, resp., compared with group A), and the expression was higher in group C compared with group B but has no statistical difference (*p* = 0.064, Figure 6(b)).

## 4. Discussions

Survival of tissue and organ depends on adequate blood supply. As long as it is for a certain period of time and degree, ischemia is bound to cause tissue damage. It is generally believed that the organ and tissue can survive and can recover completely after reperfusion as long as no irreversible damage was caused by ischemia. However, researches in recent years demonstrate that tissue and organ did not show obvious function disorder after a certain period of time of ischemia (hypoxia), while the blood reperfusion (reoxygenation) causes obvious function disorder and incurred irreversible changes. For some cells have serious ischemic injury, reperfusion did not reduce injury or recovery function but accelerated cell death.

In this study, we used an ischemia reperfusion injury model of rabbit's thigh to compare the alleviation of prefasciotomy and postfasciotomy in reperfusion injury. Porcine latissimus dorsi flap reperfusion model [19, 20] was commonly used for limb skeletal muscle ischemia reperfusion injury. However, the preparation of this model is complex, and the cost is relatively high. The cost of rat model of ischemia reperfusion injury in limb skeletal muscle is relatively low. Some researchers proposed adopting open surgery and blocking abdominal aorta or iliac artery at the beginning to block blood flow from abdominal aorta or arteria iliaca communis [21]. And some researcher proposed separating the femoral vein or femoral artery from the groin and blocking blooding flow of branch vessel using rubber tourniquet ligation and using the noninvasive vascular clamp on the femoral artery and vein to block blood flow [22]. We adopted the 2 animals' model at the preexperimental stage. However, the circulation of branch blood in lower limb muscle of the 2 animals is rich, and the produced pressure of rubber tourniquet is limited. We only observed limited injury of muscle and changes of serum enzyme after 4 hours of ischemic following reperfusion, and we did not find necrosis of muscle tissue. Saita et al. [23] prepared an ischemia reperfusion injury of skeletal muscle model in rat, but the operation is complicated and especially needs cutting thigh bone and fixating it. In this experiment, we improved the animal model, preserved the continuity of bone scaffold, cut off all the connection of skin, muscles, and nerves, and blocked the femoral vein and we could observe the necrosis of animal limb skeletal muscle, and the experimental protocol is more simple.

Zhao et al. [24] found that the myocardium of dog did not cause apoptosis in 7 hours' ischemia, while the myocardium showed obvious apoptosis after 1-hour ischemia and reperfusion for 6 hours. Myocardial cell apoptosis occurs only during reperfusion. Gottlieb et al. [25] found that 5 minutes of ischemia and then reperfusion for 4 hours and only 30 minutes and 4 or 5 hours of ischemia in isolated rabbit heart model all did not incur cardiac cell apoptosis, while 30 minutes of ischemia and 4 hours' reperfusion caused significant apoptosis of myocardial cell. These researches indicate that apoptosis is not only associated with ischemia and reperfusion, but also relevant to a certain time of ischemia plus reperfusion. Skeletal muscle is the most sensitive body tissue to ischemia. The general tolerant ischemic time of skeletal muscle is up to 4 hours. The nerve gets irreversible injury 8 hours after ischemia. The time is 13 hours for fat and 24 hours for skins. The time could be up to 4 days in bones. The damage on skeletal muscle ischemia reperfusion is the key part of limb injury, and the severity of skeletal muscle injury directly determines the severity of body's ischemia reperfusion injury.

With the prolonged time of ischemia, the changes of microcirculation lead to tissue permeability increasing and increased the swelling of tissue. The occurrence of ischemia reperfusion injury during limb replantation surgery will lead to tissue edema, increasing bone compartment pressure and compressing vascular nerve, and even cause muscle necrosis, infection, or limb necrosis. Prophylactic fasciotomy can relieve the compartment pressure, thereby effectively reducing ischemia reperfusion injury. As a product of lipid peroxidation, MDA reflect the level of oxygen radicals. Fasciotomy could reduce the infiltration of neutrophils in the injured tissue and reduced MDA means the lipid peroxidation activity is reduced, the production of lipid free radical is reduced, and the damage of oxidation product and oxidant mediated product is reduced. MPO is most abundantly expressed in neutrophil granulocytes (a subtype of white blood cells). MPO is a lysosomal protein stored in azurophilic granules of the neutrophil and released into the extracellular space during degranulation [26]. In this experiment, we demonstrated that fasciotomy could reduce the regional infiltration of neutrophils (MPO) and oxygen free radical (MDA) level in ischemic reperfusion injured muscle. Fasciotomy before the reperfusion has relative better effect compared with fasciotomy performed after the reperfusion.

Mitochondria play an important role in the survival and death of cells. Through reducing free radicals and regulating Bax and Bcl-2 expression, the opening of mitochondrial permeability transition pore could be regulated [27]. Oxygen free radicals directly cause structural damage of cells or opening of the mitochondrial permeability transition pore in ischemia reperfusion injury [28]. Fasciotomy, as an intervention for skeletal muscle ischemia reperfusion injury, can significantly reduce regional perfusion lipid oxidation degree and concentration of Ca^2+^ in mitochondrial. In this study, we found that the expression of Bax decreased and Bcl-2 increased significantly after fasciotomy, which demonstrate the effectiveness of fasciotomy for ischemia reperfusion injury.

## 5. Conclusion

In clinical work, we found that the swelling degree of muscle which received fasciotomy after reperfusion was obviously higher than fasciotomy performed before reperfusion in limb replantation. Partial limb's muscle even cannot be sutured because of severe muscle swelling when fasciotomy was performed after reperfusion. Suture or skin grafting can only be performed after swelling was alleviated after operation. Therefore, simply attributing different effects of the 2 intervention methods with the different ischemic time is not sufficient; the precise mechanism of the 2 intervention methods remains unclear. We speculate that the expandable tissue volumes for skeletal muscle are different in the 2 methods at the moment of limb blood suppling recovered, the perfusion pressures of blood are different, and the perfusion pressure reduced after fasciotomy before reperfusion. This could result in part of reason causing the difference; however, the acting mechanism of instant high flow perfusion remains to be further studied and explored. In conclusion, our research demonstrates that prefasciotomy or postfasciotomy could protect ischemia reperfusion injury in skeletal muscle. The protective effects of prefasciotomy against ischemia reperfusion injury are better than postfasciotomy for reperfusion injury. The mechanism of prefasciotomy on ischemia reperfusion injury remains to be further studied.

## References

[1] Bumbasirevic M. Z., Vuckovic C. D., Vucetic C. (2013). Replantation of upper extremity, hand and digits. *Acta Chirurgica Iugoslavica*.

[2] Zheng D., Li Z., Xu L. (2014). Application of venous flow-through flap in finger replantation with circularity soft tissue defect. *Chinese Journal of Reparative and Reconstructive Surgery*.

[3] Schmidt C. A. P., Rancic Z., Lachat M. L., Mayer D. O., Veith F. J., Wilhelm M. J. (2015). Hypothermic, initially oxygen-free, controlled limb reperfusion for acute limb ischemia. *Annals of Vascular Surgery*.

[4] Song H., Yan C., Tian X. (2017). CREG protects from myocardial ischemia/reperfusion injury by regulating myocardial autophagy and apoptosis. *Biochimica et Biophysica Acta—Molecular Basis of Disease*.

[5] Takhtfooladi H. A., Asghari A., Amirkamali S., Hoseinzadeh H. A., Takhtfooladi M. A. (2016). Evaluation of low-level laser therapy on skeletal muscle ischemia–reperfusion in streptozotocin-induced diabetic rats by assaying biochemical markers and histological changes. *Lasers in Medical Science*.

[6] Tujjar O., De Gaudio A. R., Tofani L., Di Filippo A. (2016). Effects of prolonged ischemia on human skeletal muscle microcirculation as assessed by near-infrared spectroscopy. *Journal of Clinical Monitoring and Computing*.

[7] Jawhar A., Ponelies N., Schild L. (2016). Effect of limited ischemia time on the amount and function of mitochondria within human skeletal muscle cells. *European Journal of Trauma and Emergency Surgery*.

[8] Mamedova L. K., Wang R., Besada P., Liang B. T., Jacobson K. A. (2008). Attenuation of apoptosis in vitro and ischemia/reperfusion injury in vivo in mouse skeletal muscle by P2Y6 receptor activation. *Pharmacological Research*.

[9] Park J. W., Kang J. W., Jeon W. J., Na H. S. (2010). Postconditioning protects skeletal muscle from ischemia-reperfusion injury. *Microsurgery*.

[10] Garbaisz D., Turoczi Z., Aranyi P. (2014). Attenuation of skeletal muscle and renal injury to the lower limb following ischemia-reperfusion using mPTP inhibitor NIM-811. *PLoS ONE*.

[11] Murry C. E., Richard V. J., Reimer K. A., Jennings R. B. (1990). Ischemic preconditioning slows energy metabolism and delays ultrastructural damage during a sustained ischemic episode. *Circulation Research*.

[12] Da Cunha Medeiros A., Araújo-Filho I., Tôrres M. L., De Vasconcelos Sá C., Jácome D. T., Rêgo A. C. M. (2013). Ischemic preconditioning in different times and its effect on bacterial translocation induced by intestinal ischemia and reperfusion in rats. *Revista do Colegio Brasileiro de Cirurgioes*.

[13] Bo C.-J., Chen B., Jia R.-P. (2013). Effects of ischemic preconditioning in the late phase on homing of endothelial progenitor cells in renal ischemia/reperfusion injury. *Transplantation Proceedings*.

[14] Thaveau F., Zoll J., Rouyer O. (2007). Ischemic preconditioning specifically restores complexes I and II activities of the mitochondrial respiratory chain in ischemic skeletal muscle. *Journal of Vascular Surgery*.

[15] Lintz J. A., Dalio M. B., Joviliano E. E., Piccinato C. E. (2013). Ischemic pre and postconditioning in skeletal muscle injury produced by ischemia and reperfusion in rats. *Acta Cirurgica Brasileira*.

[16] Szijártó A., Gyurkovics E., Arányi P. (2009). Effect of postconditioning in major vascular operations on rats. *Magyar sebészet*.

[17] Kuo K. K., Wu B. N., Chiu E. Y. (2013). No donor KMUP-1 improves hepatic ischemia-reperfusion and hypoxic cell injury by inhibiting oxidative stress and pro-inflammatory signaling. *International Journal of Immunopathology and Pharmacology*.

[18] Mukhopadhyay P., Horvath B., Zsengeller Z., Batkai S., Cao Z., Kechrid M. (2012). Mitochondrial reactive oxygen species generation triggers inflammatory response and tissue injury associated with hepatic ischemia-reperfusion: therapeutic potential of mitochondrially targeted antioxidants. *Free Radical Biology & Medicine*.

[19] Abdel-Rahman U., Risteski P., Klaeffling C. (2009). The influence of controlled limb reperfusion with PGE1 on reperfusion injury after prolonged ischemia. *Journal of Surgical Research*.

[20] Kaza A. K., Wamala I., Friehs I. (2017). Myocardial rescue with autologous mitochondrial transplantation in a porcine model of ischemia/reperfusion. *Journal of Thoracic and Cardiovascular Surgery*.

[21] Neto A. A. M., Júnior S. S. D. S., Capelozzi V. L. (2012). Effects of cilostazol in kidney and skeletal striated muscle of Wistar rats submitted to acute ischemia and reperfusion of hind limbs. *Acta Cirurgica Brasileira*.

[22] Zhao Z.-Q., Nakamura M., Wang N.-P. (2000). Dynamic progression of contractile and endothelial dysfunction and infarct extension in the late phase of reperfusion. *Journal of Surgical Research*.

[23] Saita Y., Yokoyama K., Nakamura K., Itoman M. (2002). Protective effect of ischaemic preconditioning against ischaemia-induced reperfusion injury of skeletal muscle: how many preconditioning cycles are appropriate?. *British Journal of Plastic Surgery*.

[24] Zhao Z.-Q., Velez D. A., Wang N.-P. (2001). Progressively developed myocardial apoptotic cell death during late phase of reperfusion. *Apoptosis*.

[25] Gottlieb R. A., Gruol D. L., Zhu J. Y., Engler R. L. (1996). Preconditioning in rabbit cardiomyocytes: role of pH, vacuolar proton ATPase, and apoptosis. *Journal of Clinical Investigation*.

[26] Wang T., Zhou Y.-T., Chen X.-N., Zhu A.-X., Wu B.-H. (2014). Remote ischemic postconditioning protects against gastric mucosal lesions in rats. *World Journal of Gastroenterology*.

[27] Qu M., Zhou Z., Chen C. (2012). Inhibition of mitochondrial permeability transition pore opening is involved in the protective effects of mortalin overexpression against beta-amyloid-induced apoptosis in SH-SY5Y cells. *Neuroscience Research*.

[28] Halladin N. L. (2015). Oxidative and inflammatory biomarkers of ischemia and reperfusion injuries. *Danish Medical Journal*.

